# Occupational therapy's role in promoting the “Learn the Signs. Act Early.” developmental monitoring program to public health employees

**DOI:** 10.1002/hcs2.95

**Published:** 2024-05-27

**Authors:** Kate Barlow, Kelsey Sullivan, Scott Lauren

**Affiliations:** ^1^ Division of Occupational Therapy American International College Springfield Massachusetts USA

**Keywords:** occupational therapy, developmental monitoring, surveillance, Head Start, “Learn the Signs. Act Early.”

## Abstract

**Background:**

Occupational therapists can play a key role in early identification of delay at the population health level by providing education to public health employees on how to implement developmental monitoring with caregivers of children birth to age 5.

**Methods:**

A pretest posttest design was utilized to assess the online education and training that was provided to Department of Public Health employees (*N* = 339), including Head Start, Special Supplemental Nutrition Program for Women, Infants and Children, Home Visiting, and Early Intervention.

**Results:**

Analysis of pretest‐posttest survey data showed significant results for all 12 key learning outcomes. Six out of 12 outcomes were found to have a large effect size (*d* > 0.8), 4 outcomes indicated a medium effect size (*d* > 0.5), and 2 outcomes had a small effect size (*d* > 0.2). Participants gained knowledge of the “Learn the Signs. Act Early.” (LTSAE) developmental monitoring program, the difference between developmental monitoring and screening, the state's referral system and age‐appropriate parental engagement activities through knowledge of child development.

**Conclusions:**

Occupational therapists are child development specialists who can provide education on developmental monitoring and activities for parental engagement. The online course proved to be an effective platform to promote LTSAE within state agencies.

## Graphical abstract

Occupational therapists can play a key role in early identification of delay at the population health level. This study evaluated the efficacy of providing online education to professionals (*N* = 339) working with children birth to 5 from Head Start, Special Supplemental Nutrition Program for Women, Infants and Children, Home Visiting, and Early Intervention on the implementation of the “Learn the Sign. Act Early.” developmental monitoring program.

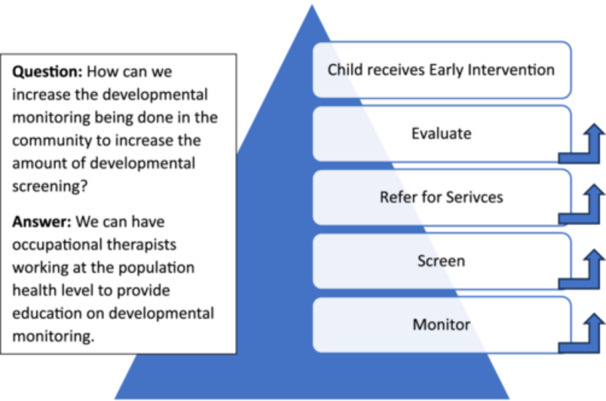

**Abbreviations**
CDC: Centers for Disease Control and Prevention
CEU: Central European University
Family TIES: Family Together in Enhancing Support
LTSAE: “Learn the Signs. Act Early.”
WIC: Women, Infants and Children

## 1 INTRODUCTION

The Centers for Disease Control and Prevention (CDC) has a free developmental monitoring program called, “Learn the Signs. Act Early.” (LTSAE). This program focuses on parental engagement for early identification of delay through recognition of developmental milestones. LTSAE developmental monitoring checklists cover social/emotional, cognitive, language/communication and motor milestones, therefore occupational therapists can help monitor a child's milestones in an area of development that an occupational therapist may not be as familiar with, such as language milestones, to make appropriate referrals when necessary. Young children who may be receiving occupational therapy services for one area of development will often have a delay in other areas [1]. Occupational therapists can utilize the LTSAE checklists in various pediatric settings, including neonatal intensive care unit follow‐up programs, early intervention home visiting programs, and in primary care [2]. The program is also helpful when children do not initially qualify for services, or as part of a home program during discharge from both inpatient and outpatient therapy services to educate parents on how to continue to monitor their child's development. Within the community setting, occupational therapists can also implement developmental monitoring for both the promotion of positive mental health [3] and for prevention and wellness as a universal approach [4].

The CDC has an Act Early Ambassador for each state as well as the territories, with larger states having two ambassadors. The Act Early Ambassador's role is to integrate developmental monitoring into state agencies, such as Head Start, the Special Supplemental Nutrition Program for Women, Infants and Children (WIC), Early Intervention and Home Visiting. From 2019 to 2023, there have been between two and three occupational therapists who serve as the CDC Act Early Ambassadors [5]. In Braveman's 2016 Health Policy Perspectives publication, he discusses the possibility of occupational therapists working for the CDC as an example of occupational therapy's role in population health, which has now become a reality. Health education, health promotion, and preventative services have already been identified as areas where occupational therapists are adequately trained to lead initiatives within communities and at the population health level [6, 7, 8, 9]. The Accreditation Council for Occupational Therapy Education [10] and the Occupational Therapy Practice Framework, 4th edition, defines occupational therapy's role as with “persons, groups, or populations” [11] and addresses a population health approach. With their training in ecological and strength‐based approaches to population health, occupational therapists are ideally suited for community health promotion programs [12]. Combined with their expertise in child development, occupational therapists can create a role in the community by identifying gaps in service delivery [8]. One of the identified gaps in service delivery is the need for developmental monitoring in the community.

### 1.1 Developmental monitoring

Developmental monitoring, one of the six parts of routine developmental surveillance completed by pediatricians, should be utilized as a supplement to developmental screening for improved identification. Unlike a screening tool, the LTSAE developmental monitoring checklists are not validated [2] and are meant to be used as communication tools to engage families in discussing their child's development. Occupational therapists are needed to complete developmental monitoring because despite the increase in developmental surveillance and screening by pediatricians since 2002 [13], only 37% of children receive developmental surveillance and 30% receive a developmental screen according to their parents [14]. In fact, it is estimated that less than half of the children with developmental disabilities in the United States who qualify actually receive early intervention services [15].

Developmental monitoring is the on‐going process of observing a child's developmental progress to see if various milestones are reached at the expected time [16]. The combination of developmental monitoring and developmental screening together has been shown to be more effective for the identification and diagnosis of delays than screening alone [17, 18]. To increase the amount of developmental monitoring that is being done in the community, occupational therapists can educate other clinicians, home visitors, and early education and care providers on how to engage parents in using the LTSAE developmental monitoring checklists. The CDC's LTSAE website (https://www.cdc.gov/ActEarly/Materials) also has other free materials, such as children's books, tip sheets for parents and clinicians and an online training program called, “Watch Me!” Use of the LTSAE program does not require any specialized training or certification, which is ideal for implementation within community settings, such as Head Start.

### 1.2 Ideal settings for developmental monitoring

The Office of Head Start is within the Administration for Children and Families under the direction of the U.S. Department of Health and Human Services and serves over one million children each year at no cost to families [19]. Head Start serves families who have fewer economic resources with children aged up to 5 years old with the primary goal of helping children achieve an adequate level of school readiness. LTSAE has previously been implemented within the Head Start settings with supportive findings [20, 21, 22]. Head Start teachers have demonstrated the commitment to caring for young children to play a key role in early identification through developmental monitoring [21].

Developmental monitoring has also been implemented within WIC programs across the state of Missouri with positive results [23]. This led to additional states, including Massachusetts, who replicated the “Missouri Model” and found a statistically significant increase in child referrals at state agencies aimed at the improvement of child health and development after implementation [24]. WIC programs provide food, education, and support to low income pregnant, breastfeeding, and nonbreastfeeding postpartum women (up to 1 year), along with infants and children up to age 5 [25].

**Figure 1:**
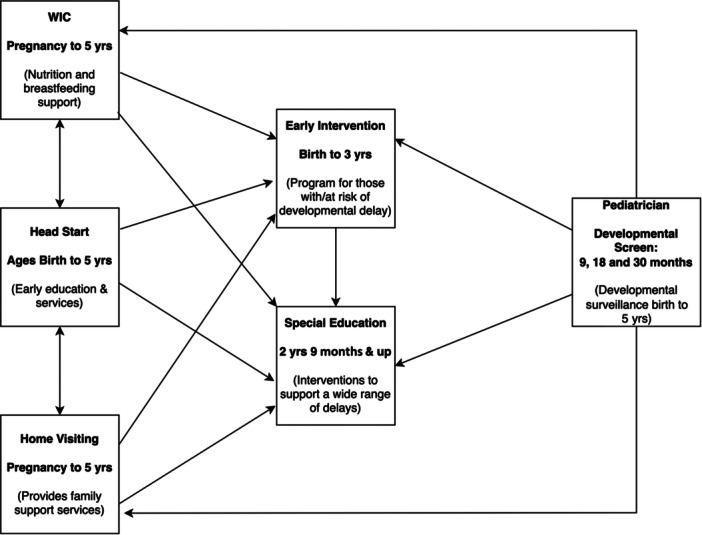
Referring agencies for children birth to 5 years.

Since Head Start and WIC programs serve low‐income families [26], and children growing up in poverty demonstrate poorer developmental outcomes [27], the implementation of developmental monitoring within these programs are a focus for CDC Act Early Ambassadors of the LTSAE program. The purpose of this study was to examine the efficacy of online training for implementation of developmental monitoring utilizing LTSAE with Department of Public Health employees working with the birth to five population, such as Head Start, WIC, Home Visiting and Early Intervention (See Figure 1). Implementation of LTSAE includes providing information on developmental monitoring through LTSAE information flyers, completing LTSAE developmental monitoring checklists with families, and utilizing other LTSAE tools, such as the children's books.

Implementation science is a method for improving outcomes for population health by implementing evidence‐based practices in public health settings [28]. This study was partially funded by the CDC and utilized both implementation science and the CDC's Framework for Program Evaluation in Public Health [29], which exemplifies the basis of implementation science. The CDC's Framework for Program Evaluation in Public Health includes six essential steps in the process including stakeholder participation, program description, design of evaluation, evidence collection, justification of findings, and disseminating conclusions [29]. This study was structured to include the necessary components of program evaluation to address the aim of this study thoughtfully and systematically.

## 2 METHODS

### 2.1 Design

Using the population health framework [30], a continuing education unit (CEU) course was purposefully created with an interprofessional lens and with stakeholder buy‐in. The principal investigator met individually with the state agencies to discuss educational and content needs, as well as access to training. The CEU course, titled, “Interprofessional Education on the Promotion of Parental Monitoring of Developmental Milestones” received approval through the state's Association for the Education of Young Children before advertising the CEU online course. Content focused on the components of the LTSAE program, such as the mobile “CDC's Milestone Tracker” app and other free resources, developmental milestones by age, tips and activities for parental engagement, and how to make referrals for screening and evaluations if necessary.

The CEU course consisted of five 1‐h classes, once a month, over the course of 5 months, using an online virtual conference software (Zoom.us). Each of the five classes were held at two different times for convenience to participants: one on a weekday evening and the other on the following weekday morning during traditional work hours. Participants could select between which of the offered times (morning or evening session) of the five classes they would like to attend.

### 2.2 Participants

This study was considered exempt through the principal investigator's institutional review board (#202015‐1). Participation in the research study was on a volunteer basis. CEU course participants were all state Public Health employees working in the birth to five population, which included Head Start (*N* = 194), WIC (*N* = 78), Home Visiting (*N* = 41), and Early Intervention (*N* = 26). Participants were recruited for the free CEU course via email from their respective state director using an electric flier with the registration link. Identifying information that was collected during registration included name, email, phone, discipline, and state agency. Other information, such as age, race, socioeconomic status, or years of experience was not collected to maintain anonymity.

### 2.3 Measures and procedure

The online course registration included a brief description of each class, as well as an explanation that each class is voluntary, however, CEUs could only be obtained by attending all five classes. Once registered, the anonymous pretest online surveys were emailed to each participant before each of the five classes. Pre‐ and postonline surveys were created by the principal investigator, which mostly included the learning objectives required for CEU's. Following completion of the presurvey, the class link was sent. During each class, attendance was documented, anonymous polls were administered, and results were recorded. After completing each class, participants also received an online anonymous posttest survey. After completion of the posttest survey, participants received a certificate of attendance for the class and a copy of the presentation slides, which included links to valuable resources, to serve as a motivating factor for completing the posttest survey.

### 2.4 Data analysis

A variety of quantitative analytical methods were used to analyze the survey data. All quantitative data was cleaned in Microsoft Excel and then analyzed using Statistical Package for the Social Sciences, version 25. For several of the pre‐ and postdata measures, a Likert scale was used resulting in categorical data on a scale of one to four. A paired *t*‐test was used to determine whether the CEU course significantly increased specific knowledge and behaviors pertaining to the subject of each class. In this case, data was first checked to ensure it met the assumptions required for the usage of this test. Therefore, the data was tested for normality using the Shapiro–Wilk test and then for equal variances using an *F*‐test. Some pre‐ and postdata was collected using binary variables. For these three variables, a new variable was created forming the sum of the three. This data was then analyzed using an independent sample *t*‐test on the sums for the individuals who attended all five classes. Finally, some questions yielded open‐ended responses. These responses were individually read and sorted into categories by keyword and then tallied to quantify the results.

## 3 RESULTS

### 3.1 Class attendance

Of the total recruited participants (*N* = 339), the total class attendance varied by session. The highest attendance recorded was in class one (*n* = 182) and lowest during class five (*n* = 89). Course sessions held during the morning were noted to have a higher attendance rate than those offered in the evenings. Courses held during the day were during regular work hours and attendance was expected to be higher. The final session was held on the Friday of a long weekend, which may have negatively impacted attendance. See Table 1 for summary of course attendance.

**Table 1:** Summary of Webinar attendance.

Attendance	Webinar 1	Webinar 2	Webinar 3	Webinar 4	Webinar 5
AM	131	100	88	108	65
PM	51	34	30	30	24
Total	182	134	118	138	89
Percentage (%)	53.69	39.53	34.81	40.71	26.25

*Note*: Total participants were 339. *N* = 339.

### 3.2 Pre–posttest survey analysis of key outcomes of course participation

Following appropriate usage of the aforementioned analytical tests, results indicated a statistically significant difference in 12 out of 12 key outcomes. Key outcomes included “ability to support developmental monitoring” (*p* = 0.001), “experience using LTSAE tools” (*p* = 0.0001), “knowledge of state agencies” (*p* = 0.0001), “ability to refer to Early Intervention” (*p* = 0.002), “ability to refer to Special Education” (*p* = 0.001), “ability to refer to Family Together in Enhancing Support (TIES)” (*p* = 0.001), “ability to describe motor milestones” (*p* = 0.001), “ability to describe cognitive milestones” (*p* = 0.001), “ability to describe social milestones” (*p* = 0.001), “ability to describe speech milestones” (*p* = 0.001), “ability to describe attachment patterns” (*p* = 0.001), and “ability to describe effects of early childhood trauma” (*p* = 0.001). The key outcomes were determined through the development of the CEU course, utilizing the primary objectives of each week's content. Effect size was calculated for the pre‐ and posttest survey and interpreted according to Cohen's criteria [31]. Conclusions included 6 out of 12 outcomes were found to have a large effect size (*d* > 0.8), 4 outcomes indicated a medium effect size (*d* > 0.5), and 2 outcomes had a small effect size (*d* > 0.2). Further breakdown of pre–posttest means, and effect sizes can be located in Table 2.

**Table 2:** Pre‐ and posttest key outcomes.

	Pre		Post				
Question	Mean	SD	Mean	SD	*t*(40)	*p* value	Cohen's *d*
Ability to support developmental monitoring	2.32	0.821	3.26	0.604	−12.178	0.0001	−1.31
Experience using LTSAE tools	1.94	0.827	3.29	0.578	−17.437	0.0001	−1.9
Knowledge of state agencies	2.3	0.781	3.15	0.656	−10.268	0.0001	−1.18
Ability to refer to Early Intervention	3.09	0.789	3.33	0.599	−3.151	0.002	−0.34
Ability to refer to Special Education	2.88	0.856	3.2	0.669	−4.18	0.001	−0.42
Ability to refer to Family TIES	2.1	0.883	3.23	0.683	−12.692	0.001	−1.43
Ability to describe motor milestones	2.74	0.616	3.15	0.541	−5.769	0.001	−0.71
Ability to describe cognitive milestones	2.79	0.674	3.13	0.508	−4.355	0.001	−0.59
Ability to describe social milestones	2.69	0.632	3.11	0.596	−5.341	0.001	−0.68
Ability to describe speech milestones	2.59	0.637	3.08	0.629	−6.047	0.001	−0.77
Ability to describe attachment patterns	2.63	0.701	3.17	0.581	7.614	0.001	−0.83
Ability to describe effects of early childhood trauma	2.63	0.701	3.21	0.58	8.604	0.001	−0.89

Abbreviations: Family TIES, Family Together in Enhancing Support; LTSAE, “Learn the Signs. Act Early”.

### 3.3 Content specific results

In addition to the key outcomes for the CEU course, the pretest‐posttest survey results revealed important information regarding participants' knowledge. Before the CEU course, 76% of the participants had not used any of the LTSAE tools. At the conclusion of the first class, researchers inquired about which developmental monitoring tool participants would try to implement first. The most common response was the CDC Milestone Tracker App (74.7%). Class one postsurvey also included the open‐ended question, “Why is it important to promote developmental monitoring?” The answers were grouped by the general theme of the response. Out of the 121 responses, 59 of participants indicated that promotion of developmental monitoring was important so that children can be identified and referred to intervention services as early as possible in hopes to reduce the future burden of care in several capacities (for the child, financially, etc.). Thirty‐five participants stated that this was important so that children could receive needed support and in turn have better outcomes. Sixteen participants responded that this was essential for parental support leading to better outcomes. Finally, 11 participants indicated that this was beneficial so an increased number of professionals would be aware of developmental monitoring and therefore more children and families would be reached by the expanded network.

Class two included questions to assess participants' knowledge and understanding of the state's referral system and other state agencies. Postsurvey results indicated a significant increase in knowledge in the state's referral process: 24% for early intervention, 29% for special education, and 67% increase for Family TIES. Family TIES is funded by the Early Intervention program and is the primary referral agency for WIC in this state. The postsurvey also asked whether Early Intervention services required a referral from a physician and 99% correctly answered “no.” Participants were similarly asked whether a physician referral was required for a special education evaluation and 86% correctly responded “no.” Participants also reported learning about a new state agencies during the session. The top 2 agencies included Family TIES (*n* = 41) and Early Intervention (*n* = 31).

Class modules three and four inquired if participants could describe three activities to promote the four domains of development in the LTSAE program. Overall, the majority of participants answered “yes” to all areas including (1) movement/physical development (97.3%), (2) cognitive (95.6%), (3) language and communication (94.4%), and (4) social and emotional (95.6%). See Table 3 for further participant answers.

**Table 3:** Participants' abilities to describe activities to promote the four key areas of development.

Area of development	Response	Participants, *n* (%)
Movement/physical	Yes	111 (97.3)
	No	3 (2.6)
Cognitive	Yes	109 (95.6)
	No	2 (1.8)
	Unsure	3 (2.6)
Language and communication	Yes	85 (94.4)
	No	1 (1.1)
	Unsure	4 (4.4)
Social and emotional development	Yes	86 (95.6)
	No	2 (2.2)
	Unsure	2 (2.2)

## 4 DISCUSSION

Approximately 40% of US children live in poverty and the pandemic has only increased the disparities, such as access to health care [32]. These children face various stressors more frequently which can impact neurologic, metabolic, and immunologic systems which ultimately can lead to poor developmental outcomes [33]. Parents' understanding of child development and how to access services also contribute to barriers in early identification [1]. The lack of an early diagnosis prevents families from accessing early intervention services which can improve outcomes for children [21]. While low‐income children historically receive fewer well‐baby/child visits and thus fewer opportunities for developmental screening [14], the Covid‐19 pandemic only further disrupted these children's abilities to receive necessary services making implementation of developmental monitoring into existing systems paramount.

Due to pandemic restrictions, this study sought to examine the efficiency of delivering developmental monitoring and implementation education in an on‐line format across the state and found it was an effective training format. The statistically significant results of the 12 key outcomes reflect enhanced knowledge of developmental milestones, referral sources, and the ability to support developmental monitoring within state agencies.

Recently published studies found that the use of LTSAE materials in Early Head Start settings facilitates parent/caregiver and staff communication as materials provide a consistent and understandable language regardless of health literacy levels [20, 22]. Understanding developmental milestones and how such materials can be utilized to engage caregivers is an essential component in the success of implementing LTSAE. Head Start professionals are better able to serve the children in their classroom if they have an understanding of child development (including social‐emotional development) as well as challenges that families may be facing in their daily lives [34].

Additionally, studies have shown that early care and education professionals have an openness to being involved in the developmental monitoring and screening process but often face barriers that limit their ability to do so [21]. Chödrön et al. [21] found the largest barrier to implementation of LTSAE was the lack of knowledge of where to refer children when there are concerns. The second training in this study's sequence was dedicated to the state's referral system and how to refer families, which may account for the participants not citing referral as one of the barriers. The LTSAE implementation barriers in Early Head Start found by Abercrombie et al. [20] also had differences, such as language barriers and parent literacy, as well as similarities, such as staff lacking the time and materials.

Limitations for this study include the lack of follow‐up for actual implementation of the LTSAE program and the postsurveys asked participants their perceived knowledge, rather than testing knowledge. Future research focused on the results of LTSAE implementation is recommended, such as the number of children referred for intervention services.

## 5 CONCLUSION

Occupational therapists working in public health can play a key role in health promotion and education utilizing a population health approach. Although there continues to be on‐going funding challenges for occupational therapists in this role [35, 36], and the CDC's Act Early Ambassador role is no exception, occupational therapists can increase their work in both public health and population health by showing the distinct value our skill set can provide. With their knowledge of child development, the referral system, and the impact of health disparities on families, occupational therapists are ideal educators and facilitators for developmental monitoring in the community. Public Health low‐income programs, like Head Start and WIC, are target locations for developmental monitoring because they serve a population of children who are at an increased risk of being adversely affected by developmental delays and disabilities. Data has shown that children who are low income, non‐White, negatively impacted by social determinants of health or adverse childhood experiences, are all at an increased risk [37]. The online continuing education course in this study was found to be an effective means to educate state agency professionals working in birth to five on the importance of developmental monitoring and its implementation to improve child outcomes.

## AUTHOR CONTRIBUTIONS

**Kate Barlow**: Conceptualization (lead); data curation (supporting); formal analysis (supporting); funding acquisition (lead); methodology (lead); project administration (lead); writing—original draft (lead); writing—review and editing (lead). **Kelsey Sullivan**: Data curation (supporting); formal analysis (supporting); methodology (supporting); writing—original draft (supporting); writing—review and editing (supporting). **Scott Lauren**: Data curation (supporting); formal analysis (supporting); project administration (supporting); writing—original draft (supporting).

## CONFLICT OF INTEREST STATEMENT

The authors declare no conflict of interest.

## ETHICS STATEMENT

This study was reviewed the American International College IRB #202015‐1 with exempt approval.

## INFORMED CONSENT

Not applicable.

## Data Availability

The data that support the findings of this study are available on request from the corresponding author. The data are not publicly available due to privacy or ethical restrictions.
